# Supplementary material for: Improved detection of SBDS gene mutation by a new method of next-generation sequencing analysis based on the Chinese mutation spectrum
Source: PLoS One. 2022 Dec 13;17(12):e0269029. doi: 10.1371/journal.pone.0269029 (PMC9747038; doi:10.1371/journal.pone.0269029)
Supplement: S2 Table — (DOCX) [file pone.0269029.s002.docx]

|  | | chr7(GRCh37):g.72301284T>C  (*SBDSP1*) | | Total | Rate |
| --- | --- | --- | --- | --- | --- |
|  |  | Positive | Negative |  |  |
| NM_016038.2:c.141C>T  (*SBDS*) | Positive | 58 | 791 | 849 | 0.0416 |
|  | Negative | 950 | 18596 | 19546 | 0.9584 |
|  | Total | 1008 | 19387 | 20395 |  |
|  | Rate | 0.0494 | 0.9506 |  |  |

There is no statistically significant relationship between two variants (p>0.05)
