# Supplementary material for: Improved detection of SBDS gene mutation by a new method of next-generation sequencing analysis based on the Chinese mutation spectrum
Source: PLoS One. 2022 Dec 13;17(12):e0269029. doi: 10.1371/journal.pone.0269029 (PMC9747038; doi:10.1371/journal.pone.0269029)
Supplement: Attachment — Submitted filename: PONE-D-21-31295.pdf [file pone.0269029.s003.pdf]

PLOS ONE

Improved detection of SBDS gene mutation by a new method of next-generation sequencing analysis based on the Chinese mutation spectrum

--Manuscript Draft--

**Manuscript Number:**	PONE-D-21-31295
**Article Type:**	Research Article
**Full Title:**	Improved detection of SBDS gene mutation by a new method of next-generation sequencing analysis based on the Chinese mutation spectrum
**Short Title:**	Improved detection of SBDS gene mutation by a new method of next-generation sequencing analysis
**Corresponding Author:**	Kaiyu Wang<br>Fulgent (Fujian) Technologies<br>Fuzhou, CHINA
**Keywords:**	
**Abstract:**	Next-generation sequencing (NGS) is a useful molecular diagnostic tool for genetic diseases. However, due to the presence of highly homologous pseudogenes, it is challenging to use short-read NGS for analyzing mutations of the Shwachman-Bodian-Diamond syndrome (SBDS) gene. The SBDS mutation spectrum was analyzed in the Chinese population, which revealed that SBDS variants were primarily from sequence exchange between SBDS and its pseudogene at the base-pair level, predominantly in the coding region and splice junction of exon two. The c.258+2T>C and c.185_184TA>GT variants were the two most common pathogenic SBDS variants in the Chinese population, resulting in a total carrier frequency of 1.19%. When analyzing pathogenic variants in the SBDS gene from the NGS data, the misalignment was identified as a common issue, and there were different probabilities of misalignment for different pathogenic variants. Here, we present a novel mathematical method for identifying pathogenic variants in the SBDS gene from the NGS data, which utilizes read-depth of the paralogous sequence variant (PSV) loci of SBDS and its pseudogene. Combined with PCR and STR orthogonal experiments, the results of SBDS gene mutation analysis were improved in 40% of clinical samples, and various types of mutations such as homozygous, compound heterozygous, and uniparental diploid were explored. The findings effectively reduce the impact of misalignment in NGS-based SBDS mutation analysis and are helpful for the clinical diagnosis of SBDS-related diseases, the research into population variation, and the carrier screening.
**Order of Authors:**	Dong Wu
	Li Zhang
	Yuzhen Qiang
	Kaiyu Wang
**Additional Information:**	
**Question**	**Response**
**Financial Disclosure**<br><br>Enter a financial disclosure statement that describes the sources of funding for the work included in this submission. Review the submission guidelines for detailed requirements. View published research articles from *PLOS ONE* for specific examples.<br><br>This statement is required for submission	The funders had no role in study design, data collection and analysis, decision to publish, or preparation of the manuscript.

Powered by Editorial Manager® and ProduXion Manager® from Aries Systems Corporation

and **will appear in the published article** if the submission is accepted. Please make sure it is accurate.<br><br>**Unfunded studies**<br>Enter: *The author(s) received no specific funding for this work.*<br><br>**Funded studies**<br>Enter a statement with the following details:<br>• Initials of the authors who received each award<br>• Grant numbers awarded to each author<br>• The full name of each funder<br>• URL of each funder website<br>• Did the sponsors or funders play any role in the study design, data collection and analysis, decision to publish, or preparation of the manuscript?<br>• **NO** - Include this sentence at the end of your statement: *The funders had no role in study design, data collection and analysis, decision to publish, or preparation of the manuscript.*<br>• **YES** - Specify the role(s) played.<br><br>* typeset	
**Competing Interests**<br><br>Use the instructions below to enter a competing interest statement for this submission. On behalf of all authors, disclose any competing interests that could be perceived to bias this work—acknowledging all financial support and any other relevant financial or non-financial competing interests.<br><br>This statement is required for submission and **will appear in the published article** if the submission is accepted. Please make sure it is accurate and that any funding sources listed in your Funding Information later in the submission form are also declared in your Financial Disclosure statement.<br><br>View published research articles from *PLOS ONE* for specific examples.	The authors have declared that no competing interests exist

Powered by Editorial Manager® and ProduXion Manager® from Aries Systems Corporation

**NO authors have competing interests**<br><br>Enter: *The authors have declared that no competing interests exist.*<br><br>**Authors with competing interests**<br><br>Enter competing interest details beginning with this statement:<br><br>*I have read the journal's policy and the authors of this manuscript have the following competing interests: [insert competing interests here]*<br><br>* typeset	
**Ethics Statement**<br><br>Enter an ethics statement for this submission. This statement is required if the study involved:<br><br>• Human participants<br>• Human specimens or tissue<br>• Vertebrate animals or cephalopods<br>• Vertebrate embryos or tissues<br>• Field research<br><br>Write "N/A" if the submission does not require an ethics statement.<br><br>General guidance is provided below. Consult the submission guidelines for detailed instructions. **Make sure that all information entered here is included in the Methods section of the manuscript.**	This study received approval from the ethics committee of No. 900 Hospital of the Joint Logistics Team.

**Format for specific study types**<br><br>**Human Subject Research (involving human participants and/or tissue)**<br>• Give the name of the institutional review board or ethics committee that approved the study<br>• Include the approval number and/or a statement indicating approval of this research<br>• Indicate the form of consent obtained (written/oral) or the reason that consent was not obtained (e.g. the data were analyzed anonymously)<br><br>**Animal Research (involving vertebrate animals, embryos or tissues)**<br>• Provide the name of the Institutional Animal Care and Use Committee (IACUC) or other relevant ethics board that reviewed the study protocol, and indicate whether they approved this research or granted a formal waiver of ethical approval<br>• Include an approval number if one was obtained<br>• If the study involved *non-human primates*, add *additional details* about animal welfare and steps taken to ameliorate suffering<br>• If anesthesia, euthanasia, or any kind of animal sacrifice is part of the study, include briefly which substances and/or methods were applied<br><br>**Field Research**<br><br>Include the following details if this study involves the collection of plant, animal, or other materials from a natural setting:<br>• Field permit number<br>• Name of the institution or relevant body that granted permission	
**Data Availability**<br><br>Authors are required to make all data underlying the findings described fully available, without restriction, and from the time of publication. PLOS allows rare exceptions to address legal and ethical concerns. See the PLOS Data Policy and FAQ for detailed information.	Yes - all data are fully available without restriction

A Data Availability Statement describing where the data can be found is required at submission. Your answers to this question constitute the Data Availability Statement and **will be published in the article**, if accepted.<br><br>**Important:** Stating 'data available on request from the author' is not sufficient. If your data are only available upon request, select 'No' for the first question and explain your exceptional situation in the text box.<br><br>Do the authors confirm that all data underlying the findings described in their manuscript are fully available without restriction?	
**Describe where the data may be found in full sentences. If you are copying our sample text, replace any instances of XXX with the appropriate details.**<br><br>• If the data are **held or will be held in a public repository**, include URLs, accession numbers or DOIs. If this information will only be available after acceptance, indicate this by ticking the box below. For example: *All XXX files are available from the XXX database (accession number(s) XXX, XXX.).*<br>• If the data are all contained **within the manuscript and/or Supporting Information files**, enter the following: *All relevant data are within the manuscript and its Supporting Information files.*<br>• If neither of these applies but you are able to provide **details of access elsewhere**, with or without limitations, please do so. For example:<br><br>*Data cannot be shared publicly because of [XXX]. Data are available from the XXX Institutional Data Access / Ethics Committee (contact via XXX) for researchers who meet the criteria for access to confidential data.*<br><br>*The data underlying the results presented in the study are available from (include the name of the third party*	All relevant data are within the manuscript and its Supporting Information files

and contact information or URL).<br>• This text is appropriate if the data are owned by a third party and authors do not have permission to share the data.<br><br>* typeset	
Additional data availability information:	

Manuscript

Click here to access/download;Manuscript;manuscript+ethics.pdf

Improved detection of *SBDS* gene mutation by a new method of next-generation sequencing analysis based on the Chinese mutation spectrum

Dong Wu[1], li Zhang[2], Yuzhen Qiang[2], Kaiyu Wang[2,†.]

Affiliations

1: Department of Obstetrics and Gynecology, 900 Hospital of the Joint Logistics Team or Dongfang Hospital, Fuzhou Fujian, People's Republic of China

2: Fulgent (Fujian) Technologies, Fuzhou Fujian, People's Republic of China

†: To whom correspondence should be addressed.

Correspondence

Kaiyu Wang, Fulgent (Fujian) Technologies; 9 Mao Ling Road, Fuzhou Fujian, People's Republic of China; 350000; [wangkaiyu@fulgent.com.cn]; +86-591-63169333.

Abstract

Next-generation sequencing (NGS) is a useful molecular diagnostic tool for genetic diseases. However, due to the presence of highly homologous pseudogenes, it is challenging to use short-read NGS for analyzing mutations of the Shwachman-Bodian-Diamond syndrome (*SBDS*) gene. The *SBDS* mutation spectrum was analyzed in the Chinese population, which revealed that *SBDS* variants were primarily from sequence exchange between *SBDS* and its pseudogene at the base-pair level, predominantly in the coding region and splice junction of exon two. The c.258+2T>C and c.185_184TA>GT variants were the two most common pathogenic *SBDS* variants in the Chinese population, resulting in a total carrier frequency of 1.19%. When analyzing pathogenic variants in the *SBDS* gene from the NGS data, the misalignment was identified as a common issue, and there were different probabilities of misalignment for different pathogenic variants. Here, we present a novel mathematical method for identifying pathogenic variants in the *SBDS* gene from the NGS data, which utilizes read-depth of the paralogous sequence variant (PSV) loci of *SBDS* and its pseudogene. Combined with PCR and STR orthogonal experiments, the

results of *SBDS* gene mutation analysis were improved in 40% of clinical samples, and various types of mutations such as homozygous, compound heterozygous, and uniparental diploid were explored. The findings effectively reduce the impact of misalignment in NGS-based *SBDS* mutation analysis and are helpful for the clinical diagnosis of *SBDS*-related diseases, the research into population variation, and the carrier screening.

1. Introduction

High-throughput, high-sensitivity, and low-cost next-generation sequencing (NGS) have made tremendous progress in the field of clinical molecular testing. Analysis with NGS accurately obtains information on various types of mutation by repeatedly reading the sequence of each base and searching for the optimum matching coordinates in the reference genome [1]. However, when a gene region has one or more pseudogenes with high sequence homology, the short NGS reads cannot be aligned accurately to the target locus, which, in turn, increases the likelihood of false positives or false negatives [2]. Misalignment is more likely to occur, especially when there is a conversion between gene and its pseudogene, presented by the exchange of one or multiple paralogous sequence variants (PSVs) [3]. Therefore, the interference caused by pseudogenes as well as their conversion is one of the key problems with the clinical application of NGS.

The protein encoded by the Shwachman-Bodian-Diamond syndrome (*SBDS*) gene is involved in ribosomal RNA processing. Since its first publication in 2003, pathogenic variants in the *SBDS* gene have been detected in more than 90% of people with Shwachman-Diamond syndrome (SDS) [4]. Also known as Shwachman-Bodian-Diamond syndrome, SDS is an autosomal recessive genetic disease involving multiple systems. Its clinical manifestations include exocrine pancreatic dysfunction (chronic diarrhea), skeletal metaphysis development disorder, and different degrees of bone marrow dysfunction accompanied by hypocytosis [5, 6]. About one-third of patients suffer from myelodysplastic syndrome and acute myeloid leukemia [7]. Other clinical symptoms include short stature, liver dysfunction, susceptibility to infection, nephrocalcinosis, myocardial necrosis, and neonatal respiratory distress [8, 9]. Dozens of *SBDS* pathogenic variants have been reported to cause SDS, the most common of which include c.185_184TA>GT, c.258+2T>C, and c.[185_184TA>GT;258+2T>C]. A study used targeted analysis for the three common *SBDS* pathogenic variants and detected at least one pathogenic variant in 90% of affected individuals and

both pathogenic variants in approximately 62% of affected individuals who had SDS along with *SBDS* [4]. Nonetheless, multi-gene testing using NGS is still recommended in the molecular diagnosis of suspected cases of SDS that can be caused by pathogenic variants in other genes besides *SBDS*. Additionally, the phenotypic spectrum of SDS is broad, and multi-gene testing might be the best option for an individual with atypical phenotypic features [10].

The *SBDS* gene has a highly homologous pseudogene, *SBDSP1*, which is located ~ 5.8Mb downstream of the *SBDS* gene at chromosome 7q11. The five exon coding sequences of *SBDS* have 96.8% (95%–98.2%) homology with *SBDSP1*. Since the common *SBDS* pathogenic variants c.185_184TA>GT and c.258+2T>C are at the functional PSV loci, it has been suggested that recombination and gene conversion might occur between *SBDS* and *SBDSP1* [11]. Based on the above factors, researchers have recognized the complexity and challenges of NGS analysis of the *SBDS* gene, and therefore suggest using PCR, RT-PCR or specific bioinformatics tools to increase the sensitivity and specificity of detecting pathogenic variants in *SBDS* [12-14].

This study focused on analyzing the *SBDS* mutation spectrum through large-scale whole-exome sequencing (WES) within the Chinese population. After determining the misalignment issues in the NGS data analysis, we tried to overcome these problems by developing a novel mathematical method using NGS data to analyze the pathogenic variants of the *SBDS* gene.

2. Materials And Methods

DNA Samples

To study the *SBDS* mutation spectrum of the Chinese population and the misalignment of NGS data, we selected 20,542 de-identified samples that were sent to Fulgent (Fujian) Technologies Co., Ltd.(Short for “Fujian Fulgent”) for WES by NGS from October, 2019 to March, 2021. Authors could not access to information that could identify individual participants during or after data collection after application. All research participants were from the Chinese population, and their EDTA anti-coagulated venous whole blood was obtained after signing their informed consent. Samples were divided into non-SDS and suspected SDS groups according to whether the individual’s clinical diagnosis included SDS phenotypes, such as chronic diarrhea, cytopenia, or liver dysfunction. The non-SDS group included a total of 20,395 cases, in which none of the

above phenotypes were detected. The suspected SDS group contained any two of the above phenotypes and included a total of 147 cases. Since most of the samples sent to Fujian Fulgent were from suspected cases of genetic diseases, the grouping criteria did not include common phenotypes of genetic diseases, such as growth failure and bone abnormalities, but included liver dysfunction, which is reportedly more common in people from China with SDS. According to the manufacturer's instructions, the genomic DNA was extracted from whole blood using commercially available DNA isolation kits. This study received approval from the ethics committee of No. 900 Hospital of the Joint Logistics Team.

Whole-Exome Sequencing

Genomic DNA samples were analyzed using the Illumina HiSeq or NovaSeq platforms (Illumina, USA) with PE150 (paired-end sequencing, 150-bp reads). Briefly, DNA libraries were prepared using the IDT xGen Exome Research Panel v2 exome library (71Mb target region). Samples from the same capture pool were grouped together. The raw sequence data were aligned to the human reference genome GRCh37/hg19 using Novoalign v3.09.00 software (Novocraft Technologies, Malaysia). SNPs and short indels were called using SAMtools v1.3.1 and VarScan software v2.3.9. Variants were annotated using Alamut-Batch standalone v1.9 software (Interactive Biosoftware, France). The average read-depth of each exon of *SBDS* and the corresponding region of *SBDSP1*, as well as the read-depth of their PSVs loci, were extracted using bedtools coverage (v.2.30.0). Variants were curated manually according to the American College of Medical Genetics (ACMG) scoring system [15].

Sanger sequencing

The primer sequences provided by Woloszynek were employed to amplify the five exons, splice sites, and intron sequences flanking the exons of *SBDS* [16]. Amplicons were generated using 5 ng of genomic DNA using Ex Taq polymerase (Takara, China). The PCR conditions for this amplification were as follows: 95 °C for 5 min, 35× (95 °C for 30 s and 60 °C for 15 min), 72 °C for 5 min. Products were verified by agarose gel electrophoresis then sequenced using the sequencing primers on a 3730 genetic analyzer (Applied Biosystems, USA).

STR typing

In the parentage test, samples DNA were detected with Microreader™ 21 ID System (Suzhou Microread Genetics, Suzhou, China), according to the standard procedures of the manufacturer.

3. Results and Discussions

Previous studies have speculated that *SBDS* pathogenic variants may be converted from *SBDSP1* because these pathogenic variants often occur at the functional PSV loci of *SBDS* and sometimes include other cis variants at PSV loci [11]. Therefore, research has sought to determine if the formation of *SBDS* pathogenic variants occurs through gene conversion and how frequently this conversion takes place. This information is very important for formulating NGS data analysis strategies for *SBDS* genes and validating the accuracy of the results [2]. This study aimed to acquire this information through large-scale WES NGS data analysis. In general, the average read-depth ratio of a gene to its pseudogene will significantly shift in genes or regions where gene conversion is more frequent, such as exons 13–15 of the *PMS2* gene [17]. The average read-depths of each exon of *SBDS* and the corresponding region of *SBDSP1* were extracted to estimate the frequency of conversion between *SBDS* and *SBDSP1*. Subsequently, the read-depth ratios of *SBDS* to *SBDSP1* were calculated of 2000 WES samples, which were randomly selected from the Chinese population in the non-SDS group. Theoretically, when *SBDS* is converted to *SBDSP1*, the read-depth of *SBDS* becomes lower than that of the corresponding *SBDSP1* region, thereby leading to a decrease in the ratio of *SBDS* to *SBDSP1*. When the opposite conversion occurs, the ratio increases. In this study, the average read depth of each exon of *SBDS* was not significantly different from that of the corresponding region of *SBDSP1* (Figure 1), indicating that the *SBDS* NGS data can usually be accurately aligned, while gene conversion of *SBDS* and *SBDSP1* in exon-level or in a larger range is not common.

Further analysis aimed to determine if the interlocus conversion between the *SBDS* and *SBDSP1* genes occurred at the sub-exon level [18, 19]. Since this conversion was manifested as the exchange of PSVs in NGS analysis, we tried to obtain the *SBDS* mutation spectrum from WES data of 20,395 samples from the Chinese population. A total of 64 different *SBDS* variants were discovered, of which seven were detected more than ten times (Supplement Table 1). Among them, c.141C>T, c.258+2T>C, and c.201A>G were the top three most frequently detected variants, with carrier frequencies of 4.16%, 1.01%, and 0.61%, respectively, and all were at PSV loci of *SBDS*. The total carrier frequency of ten variants at PSV loci of *SBDS* was 6.18%, which was significantly higher than other fifty-four at non-PSV position (1.23%). In addition, through

analyzing the NGS data of *SBDSP1*, the frequency of these common variants in *SBDS* were found to vary in accordance with variants in the corresponding *SBDSP1* loci (P < 0.05), but did not results from the reciprocal sequence exchanges because there was no significant correlation between their occurrence (P>0.05) (Supplement Table 2). This observation indicated that the variants of *SBDS* mainly originated from the gene-conversion event, the frequency of which was very low in the Chinese population. Furthermore, we observed that most variants at PSV loci of *SBDS* were detected in isolation, and only 35 samples were found to have in-cis variants at adjacent PSV loci, all of which were in exon two and its flanking sequence of the *SBDS* gene. According to the distance between PSVs in the *SBDS* coding region, it can be inferred that sequence conversions between *SBDS* and *SBDSP1* mainly occurred at the base pairs level, and longer conversions at the sub-exon level only occur in the Exon two region.

In order to analyze the pathogenic variant spectrum of *SBDS* in the Chinese population and provide a basis for the clinical diagnosis and carrier screening of SDS, we classified the pathogenicity of 64 different variants in 20,395 samples according to the 2015 ACMG guidelines. Four variants were found that could be classified as pathogenic or likely pathogenic. The c.258+2T>C variant was detected most frequently (207 cases), followed by c.185_184TA>GT (33 cases), while the other two were null mutations which were only detected once (Table 1 and Supplement Table 1). The total carrier frequency of *SBDS* pathogenic variants in the Chinese population was 1.19%. According to Hardy-Weinberg equilibrium, the theoretical incidence of *SBDS*-related SDS in the Chinese population was 3.52 per 100,000, which was higher than that of other populations (0.5-1.5/100,000) [20]. This is the first study to analyze the carrier frequency of *SBDS* pathogenic variants in the Chinese population with large sample size. SDS is a severe early-onset disease, and the overall carrier frequency of *SBDS* gene pathogenic variants in the Chinese population is >1% [21]. Therefore, this study provided evidence for the *SBDS* gene as a candidate for screening potential carriers. Regarding the spectrum of pathogenic variants in the population, analyzing the common hotspot mutations, such as c.258+2T>C and c.185_184TA>GT, could effectively screen out most of the carriers.

Since PSVs could be informative for NGS read alignment for highly homologous genes, the more variants at *SBDS* PSVs loci occurred in a given NGS read, the more likely it was to be

misaligned to *SBDSP1*, and the higher was the risk of false negatives. In the NGS data with 207 c.258+2T>C and 33 c.185_184TA>GT, it was found that most c.258+2T>C were detected in isolation (91.3%), and only a few detected *in-cis* with a variant at the c.201A PSV (8.7%). On the other hand, c.185_184TA>GT had a higher proportion (51.5%) detected *in-cis* with variants at the c.141C and/or c.201A PSVs, suggesting that NGS data reads containing c.185_184TA>GT have a greater chance of misalignment (table). In our study, the allele balance of c.258+2T>C and c.185_184TA>GT both had a significant shift. Especially, nearly half of c.185_184TA>GT variants had allele balance < 0.3, the majority of which were arranged *in-cis* with other variants at PSV loci (Figure 2). Allele balance bias made it difficult to distinguish whether the mutation reads were chimeric or misaligned. Furthermore, allele balance bias is an important quality control filter of NGS data. A pathogenic variant might be filtered if the allele balance is below the limit (usually 10%). Consequently, misdiagnosis and missed diagnosis were likely to occur, especially for individuals with atypical SDS symptoms. These two pathogenic variants were even excluded from The Exome Aggregation Consortium (ExAC) database due to failure to pass the variant quality score recalibration (VQSR) filter. It is worth noting that the common c.[185_184TA>GT; 258+2T>C] variant was not found in the Chinese population [22], which was inconsistent with the theoretically predicted carrier frequency. It was speculated that the *SBDS* NGS reads containing this variant were completely aligned to the *SBDSP1* locus due to the exchange of multiple PSVs, suggesting that the NGS analysis on c.[185_184TA>GT;258+ 2T>C] had limitations. Hence, it is necessary to establish a more effective analysis process to resolve the interference caused by the misalignment of NGS data in the analysis of *SBDS* variation.

In view of the above analysis of the *SBDS* variant and pathogenic variant spectrum in the Chinese population, we have found that the pathogenic variants of *SBDS* were mainly at functional PSV loci caused by short sequence exchange in the coding region and splice junction of exon two. Therefore, we formulated an NGS data processing procedure for *SBDS* in this particular region to overcome the NGS misalignment problem. The primary strategy comprised the generation of a mathematical relationship between the *SBDS*:*SBDSP1* read-depth ratio (RR) of each PSV locus and the total *SBDS*+*SBDSP1* read-depth (TR) of each PSV locus. As shown in Figure 3, with wild-type *SBDS* and *SBDSP1*, NGS reads could be accurately aligned to the correct

locus, and the RR values of all PSV loci were about 1 (between 0.8-1.2). When one or multiple PSVs of *SBDSP1* appeared in *SBDS* reads, a portion of *SBDS* reads was misaligned to *SBDSP1*, and the RR values of PSV loci were reduced (< 1). At this time, variants with a low allele balance could be called at PSV loci of the *SBDS* gene, and variants with a low allele balance adjacent to the corresponding PSV loci of *SBDSP1* could also be called. The TR value of PSVs loci was not significantly different from that of other WES data in the same capture pool. When there were more adjacent PSVs of *SBDSP1* in *SBDS* reads, more *SBDS* reads were misaligned to *SBDSP1*, and the RR values of multiple PSV loci decreased (as low as 1/3). We could not identify mutations in *SBDS* or *SBDSP1*, but the TR value of PSVs loci was not significantly different from that of other WES data in the same capture pool. In addition, when the individual carrying a heterozygous deletion of the *SBDS* gene, the RR values of multiple PSV loci were also reduced (~1/2), but the TR value was lower than that of other WES data in the same capture pool, which was different from the misalignment. The novel strategy had the potential to find misalignment events in *SBDS* NGS analyses and provides an additional opportunity to manually check NGS data and apply orthogonal methods.

Ten homozygous or compound heterozygous pathogenic variants in the *SBDS* gene were detected from WES data of 147 cases in the suspected SDS group using the above *SBDS* NGS analysis strategy. Among them, c.258+2T>C had the highest number of detections (10 cases/100%), followed by c.185_184TA>GT (5 cases per 50%), c.[185_184TA>GT;258+2T>C] (1 case/10%) and c.120del (1 case/10%); three homozygous pathogenic variants were all c.258+2T>C. Our analysis strategy improved the sequencing results of 40% (4/10) of the samples.

In one case (case 1), a c.258+2T>C variant in homozygous form was initially identified in the general NGS analysis. Further comparison with other WES data in the same capture pool, reduced RR values (approximately 1/3) and normal TR values were found at multiple PSV loci across exon two of *SBDS* gene, which indicated a potential misalignment event in the NGS analysis (Figure 4 and 5). Post-verification by Sanger sequencing, the case was ultimately proved to be a compound heterozygote of c.258+2T>C and c.[141C>T;185_184TA>GT;201A>G;258+2T>C] (Figure 5). The reason for the false homozygosity result was that NGS reads containing *SBDS* variants c.[141C>T;185_184TA>GT;201A>G;258+2T>C] were all misaligned to the *SBDSP1* locus.

In two cases, a c.258+2T>C variant in heterozygous form was initially identified in general NGS analysis. Further comparison with other WES data in the same capture pool, reduced RR values, and normal TR values were found at multiple PSV loci in exon two of *SBDS* gene, which indicated a potential misalignment event in the NGS analysis. For example, in one representative case (case 2), the c.185_184TA>GT and c.201A>G variants were found in a few reads (3/35) at the *SBDS* PSV loci when we further manually checked the BAM file, which was not identified because they were filtered out by the mapping quality threshold (10%). At the same time, the n.489T>C variant was found at the *SBDSP1* PSV locus, which might have been misaligned from the NGS reads of *SBDS* (Figure 6 and 7). Post-verification by Sanger sequencing, the case was ultimately proved to be a compound heterozygote of c.258+2T>C and c.[185_184TA>GT; c.201A>G] (Figure 7). Thus, our strategy for *SBDS* NGS analyses provided an additional opportunity to manually check NGS data, which could be helpful for finding and distinguishing the authenticity of variants with extreme allele balances.

Due to the misalignment of NGS data, *SBDS* variants sometimes have abnormal allele balances, which would then manifest as unexpected segregation patterns or be misunderstood as somatic mosaicism. However, abnormal genetic events like these are really rare in reported cases. This was also supported by the fact that all *SBDS* variants from the suspected SDS group were inherited from heterozygous parents, proved by orthogonal methods. In particular, we found that one case (case 3) had a homozygous c.258+2T>C variant only inherited from a heterozygous father. The WES data showed normal RR and TR values at the *SBDS* PSV loci, which ruled out the possibility of large fragment DNA deletion or reads misalignment in the *SBDS* gene (Figure 8 and 9). Further, a multiplex STR testing was done to explain this unexpected pattern of segregation, and the results showed that the homozygous c.258+2T>C variant was caused by the paternal uniparental disomy of chromosome seven (Figure 10). This is the first report of uniparental disomy caused a homozygous pathogenic variant in SDBS. It is suggested that our strategy for *SBDS* NGS analyses provided an additional opportunity to apply more appropriate orthogonal methods.

4. Conclusion

Our proposed NGS variant data analysis method, based on read-depth analysis of the PSV

loci of *SBDS* and its pseudogene, could effectively overcome the misalignment problem in *SBDS* NGS data analysis and improve detection rates of *SBDS* pathogenic variants. Based on the Chinese mutation spectrum of *SBDS*, the novel method not only focused on the most common variants in the *SBDS* gene exon 2, but also could be used for the detection of structural variation (SV) in *SBDS*. Furthermore, the method was useful for performing more efficient molecular diagnosis, the variant spectrum study, and large-scale carrier screening of SDS.

Reference

1. Rehder C, Bean LJH, Bick D, Chao E, Chung W, Das S, O'Daniel J, Rehm H, Shashi V, Vincent LM; ACMG Laboratory Quality Assurance Committee. Next-generation sequencing for constitutional variants in the clinical laboratory, 2021 revision: a technical standard of the American College of Medical Genetics and Genomics (ACMG). Genet Med. 2021. 23(8):1399-1415. doi: 10.1038/s41436-021-01139-4.
2. Lee CY, Yen HY, Zhong AW, Gao H. Resolving misalignment interference for NGS-based clinical diagnostics. Hum Genet. 2021. 140(3):477-492. doi: 10.1007/s00439-020-02216-5.
3. Blasco-Pérez L, Paramonov I, Leno J, Bernal S, Alias L, Fuentes-Prior P, Cuscó I, Tizzano EF. Beyond copy number: A new, rapid, and versatile method for sequencing the entire SMN2 gene in SMA patients. Hum Mutat. 2021. 42(6):787-795. doi: 10.1002/humu.24200.
4. Boocock GR, Morrison JA, Popovic M, Richards N, Ellis L, Durie PR, Rommens JM. Mutations in SBDS are associated with Shwachman-Diamond syndrome. Nat Genet. 2003. 33(1):97-101. doi: 10.1038/ng1062.
5. Ginzberg H, Shin J, Ellis L, Morrison J, Ip W, Dror Y, Freedman M, Heitlinger LA, Belt MA, Corey M, Rommens JM, Durie PR. Shwachman syndrome: phenotypic manifestations of sibling sets and isolated cases in a large patient cohort are similar. J Pediatr. 1999. 135(1):81-8.
6. Cipolli M, D'Orazio C, Delmarco A, Marchesini C, Miano A, Mastella G. Shwachman's syndrome: pathomorphosis and long-term outcome. J Pediatr Gastroenterol Nutr. 1999. 29(3):265-72.
7. Donadieu J, Fenneteau O, Beaupain B, Beaufils S, Bellanger F, Mahlaoui N, Lambilliotte A, Aladjidi N, Bertrand Y, Mialou V, Perot C, Michel G, Fouyssac F, Paillard C, Gandemer V, Boutard P, Schmitz J, Morali A, Leblanc T, Bellanné-Chantelot C; Associated investigators of the French Severe Chronic Neutropenia Registry. Classification of and risk factors for hematologic complications in a French national cohort of 102 patients with Shwachman-Diamond syndrome. Haematologica. 2012. 97(9):1312-9. doi: 10.3324/haematol.2011.057489.
8. Mäkitie O, Ellis L, Durie PR, Morrison JA, Sochett EB, Rommens JM, Cole WG. Skeletal phenotype in patients with Shwachman-Diamond syndrome and mutations in SBDS. Clin Genet. 2004 Feb;65(2):101-12. doi: 10.1111/j.0009-9163.2004.00198.x. PMID: 14984468.
9. Toiviainen-Salo S, Durie PR, Numminen K, Heikkilä P, Marttinen E, Savilahti E, Mäkitie O. The natural history of Shwachman-Diamond syndrome-associated liver disease from

childhood to adulthood. J Pediatr. 2009 Dec;155(6):807-811.e2. doi: 10.1016/j.jpeds.2009.06.047. Epub 2009 Aug 14. PMID: 19683257.

10. Ye Z, Zhou Y, Huang Y, Sun H, Wang H, Wu B. Clinical Characteristics and Genetic Causes of Infantile Exocrine Pancreatic Insufficiency in Chinese Patients: Study From a Tertiary Care Center. Pancreas. 2018 Nov/Dec;47(10):1344-1349. doi: 10.1097/MPA.0000000000001185. PMID: 30308536.
11. Nakashima E, Mabuchi A, Makita Y, Masuno M, Ohashi H, Nishimura G, Ikegawa S. Novel SBDS mutations caused by gene conversion in Japanese patients with Shwachman-Diamond syndrome. Hum Genet. 2004 Mar;114(4):345-8. doi: 10.1007/s00439-004-1081-2. Epub 2004 Jan 29. PMID: 14749921.
12. Yamada M, Uehara T, Suzuki H, Takenouchi T, Inui A, Ikemiyagi M, Kamimaki I, Kosaki K. Shortfall of exome analysis for diagnosis of Shwachman-Diamond syndrome: Mismapping due to the pseudogene SBDSP1. Am J Med Genet A. 2020 Jul;182(7):1631-1636. doi: 10.1002/ajmg.a.61598. Epub 2020 May 15. PMID: 32412173.
13. Jia H, Guo Y, Zhao W, Wang K. Long-range PCR in next-generation sequencing: comparison of six enzymes and evaluation on the MiSeq sequencer. Sci Rep. 2014 Jul 18;4:5737. doi: 10.1038/srep05737. PMID: 25034901; PMCID: PMC4102922.
14. Xiaomin Peng, Xinran Dong, Yaqiong Wang, Bingbing Wu, Huijun Wang, Wei Lu, Feifan Xiao, Lin Yang, Gang Li, Wenhao Zhou, Bo Liu, Yulan Lu. 2021. Overcoming the pitfalls of NGS-based molecular diagnosis of Shwachman-Diamond syndrome. doi: https://doi.org/10.1101/2021.08.22.21262444.
15. Richards S, Aziz N, Bale S, Bick D, Das S, Gastier-Foster J, Grody WW, Hegde M, Lyon E, Spector E, Voelkerding K, Rehm HL; ACMG Laboratory Quality Assurance Committee. Standards and guidelines for the interpretation of sequence variants: a joint consensus recommendation of the American College of Medical Genetics and Genomics and the Association for Molecular Pathology. Genet Med. 2015. 17(5):405-24. doi: 10.1038/gim.2015.30.
16. Woloszynek JR, Rothbaum RJ, Rawls AS, Minx PJ, Wilson RK, Mason PJ, Bessler M, Link DC. Mutations of the SBDS gene are present in most patients with Shwachman-Diamond syndrome. Blood. 2004. 1;104(12):3588-90. doi: 10.1182/blood-2004-04-1516.
17. Li J, Dai H, Feng Y, Tang J, Chen S, Tian X, Gorman E, Schmitt ES, Hansen TA, Wang J, Plon SE, Zhang VW, Wong LJ. A Comprehensive Strategy for Accurate Mutation Detection of the Highly Homologous PMS2. J Mol Diagn. 2015. 17(5):545-53. doi: 10.1016/j.jmoldx.
18. Sudmant PH, Kitzman JO, Antonacci F, Alkan C, Malig M, Tsalenko A, Sampas N, Bruhn L, Shendure J; 1000 Genomes Project, Eichler EE. Diversity of human copy number variation and multicopy genes. Science. 2010. 29;330(6004):641-6. doi: 10.1126/science.1197005.
19. Vollger MR, Dishuck PC, Sorensen M, Welch AE, Dang V, Dougherty ML, Graves-Lindsay TA, Wilson RK, Chaisson MJP, Eichler EE. Long-read sequence and assembly of segmental duplications. Nat Methods. 2019 Jan;16(1):88-94. doi: 10.1038/s41592-018-0236-3. Epub 2018 Dec 17. PMID: 30559433; PMCID: PMC6382464.
20. Minelli A, Nicolis E, Cannioto Z, Longoni D, Perobelli S, Pasquali F, Sainati L, Poli F, Cipolli M, Danesino C. Incidence of Shwachman-Diamond syndrome. Pediatr Blood Cancer. 2012. 15;59(7):1334-5. doi: 10.1002/pbc.24260.

21. Committee Opinion No. 690 Summary: Carrier Screening in the Age of Genomic Medicine. Obstet Gynecol. 2017 Mar;129(3):595-596. doi: 10.1097/AOG.0000000000001947.
22. An WB, Liu C, Wan Y, Chang LX, Chen XY, Zhu XF. [Clinical features and gene mutations of children with Shwachman-Diamond syndrome and malignant myeloid transformation]. Zhongguo Dang Dai Er Ke Za Zhi. 2020. 22(5):460-465. Chinese. doi: 10.7499/j.issn.1008-8830.2001133.

Table 1 Pathogenic or likely pathogenic variants detected *SBDS* gene in non-SDS group

HGVS	Protein	N[a]	Frequency	Classification	Evidence
c.258+2T>C	p.?	207	1.01%	Pathogenic	PVS1, PM3_Strong
c.183_184delTAinsCT	p.Lys62*	33	0.16%	Pathogenic	PVS1, PS3, PM3_Strong
c.18dupC	p.Thr7Hisfs*43	1	0.00%	Likely Pathogenic	PVS1, PM2_Supporting
c.624+1G>A	p.?	1	0.00%	Likely Pathogenic	PVS1, PM2_Supporting

a The number of variant carriers

Table 2 The arrangement of variants detected at PVS loci of exon 2 region of *SBDS* gene

<table>
<tr><th>258+2T>C</th><th>201A>G</th><th>185_184TA>GT</th><th>c.141C>T</th><th>N[a]</th><th>Percentage</th></tr>
<tr><td rowspan="2">+</td><td>-</td><td>-</td><td>-</td><td>199</td><td>96.14%</td></tr>
<tr><td>+</td><td>-</td><td>-</td><td>18</td><td>8.70%</td></tr>
<tr><td>-</td><td>-</td><td rowspan="4">+</td><td>-</td><td>16</td><td>48.48%</td></tr>
<tr><td>-</td><td>+</td><td>-</td><td>11</td><td>33.33%</td></tr>
<tr><td>-</td><td>-</td><td>+</td><td>3</td><td>9.09%</td></tr>
<tr><td>-</td><td>+</td><td>+</td><td>3</td><td>9.09%</td></tr>
</table>

a The number of variant carriers in non-SDS group

FIGURE 1 The Misalignment analysis of *SBDS* to detect gene conversion. The red lines are the expected the read-depth ratio values when the conversion between *SBDS* and *SBDSP1* occurs. The median read-depth ratio of *SBDS* to *SBDSP1* among 2000 random samples in non-SDS group. The error bars represent the standard error of the median read-depth ratio.

FIGURE 2 Allele balance of *SBDS* gene pathogenic variants c.258+2T>C (n=207) and c.185_184TA>GT (n=33) in non-SDS group. Note the y-axis for the number of heterozygous variants carriers. Variants with low allele balance (<0.3) are considered to be having misalignment issue.

FIGURE 3 Overview of misalignment caused by variants at the coding region and splice junction of exon two of *SBDS* gene. In this illustration, there are five nucleotide differences between *SBDS* and *SBDSP1*. When one or multiple variants occur at PSV loci, alignment programs will map the reads to the wrong position. A. Wild-type *SBDS* gene with correct mapping result. B. *SBDS* gene with a heterozygous variant (c.185_184TA>GT) at PSV loci, which will be called in *SBDS* gene with low allele balance. C. *SBDS* gene with multiple *in-cis* heterozygous variants at PSV loci, which will not be called in *SBDS* gene. D. *SBDS* gene with large heterozygous deletion.

FIGURE 4 Read-depths of the four genomic positions at the PSV loci of *SBDS* gene and the corresponding loci of *SBDSP1* gene (case 1). The bars with a dashed outline represent the read-depths at the loci of *SBDS* gene; the adjacent bars with a dotted outline represent the read-depths at the corresponding loci of *SBDSP1* gene. Control represent the mean read-depth of other WES data in the same capture pool.

FIGURE 5 Aligned NGS reads from WES data (top) and Sanger sequencing peak map (bottom) of case 1. NGS reads are shown by the IGV software using genomic coordinates. The WES data showed that case 1 was homozygous for the 258+2T>C variant. The read-depth of exon 2 region of *SBDS* gene is reduced, and the read-depth of the corresponding region of *SBDSP1* gene is increased. Sanger sequencing results indicated that case 1 was a compound heterozygote for c.258+2T>C and c.[141C>T;183_184TA>GT;201A>G;258+2T>C].

FIGURE 6 Read-depths of the four genomic positions at the PSV loci of *SBDS* gene and the corresponding loci of *SBDSP1* gene (case 2). The bars with a dashed outline represent the read-depths at the loci of *SBDS* gene; the adjacent bars with a dotted outline represent the read-depths at the corresponding loci of *SBDSP1* gene. Control represent the mean read-depth of other WES data in the same capture pool.

FIGURE 7 Aligned NGS reads from WES data (top) and Sanger sequencing peak map (bottom) of case 2. NGS reads are shown by the IGV software using genomic coordinates. The WES data showed that case 2 was homozygous for the 258+2T>C variant, and reads with 201A>G and 183_184TA>CT were disregarded as low-quality reads. The read-depth of exon 2 region of *SBDS* gene is reduced and the read-depth of the corresponding region of *SBDSP1* gene is increased. Sanger sequencing results indicated that case 2 was a compound heterozygote for c.258+2T>C and c.[183_184TA>GT;201A>G].

FIGURE 8 Read-depths of the four genomic positions at the PSV loci of *SBDS* gene and the corresponding loci of *SBDSP1* gene (case 3). The bars with a dashed outline represent the read-depths at the loci of *SBDS* gene; the adjacent bars with a dotted outline represent the read-depths at the corresponding loci of *SBDSP1* gene. Control represent the mean read-depth of other WES data in the same capture pool.

FIGURE 9 Aligned NGS reads from WES data of case 3. NGS reads are shown by the IGV software using genomic coordinates. The WES data showed that case 3 was homozygous for the 258+2T>C variant. The read-depth of exon 2 region of *SBDS* gene had no significant difference from that of the corresponding region of *SBDSP1* gene.

FIGURE 10 STR analysis result of chromosome 7 (case 3 family). STR analysis showed that all STR polymorphisms of case 3 are inherited from both parents, expect the STR polymorphisms of chromosome 7 are proved to be paternally inherited.

Figure 1

Click here to access/download;Figure;file-1.tif

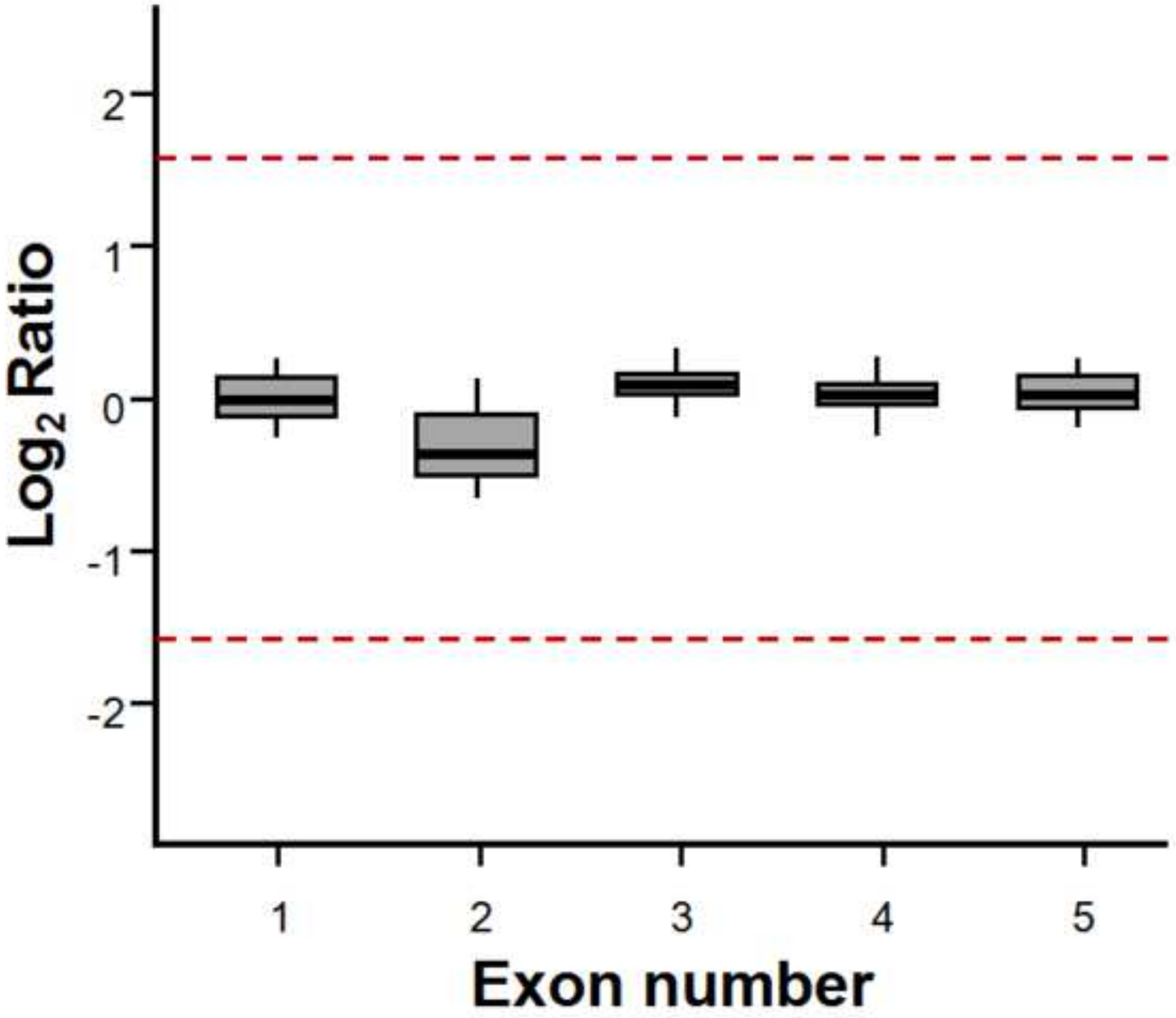

Figure 2

Click here to access/download;Figure;file-2.tif

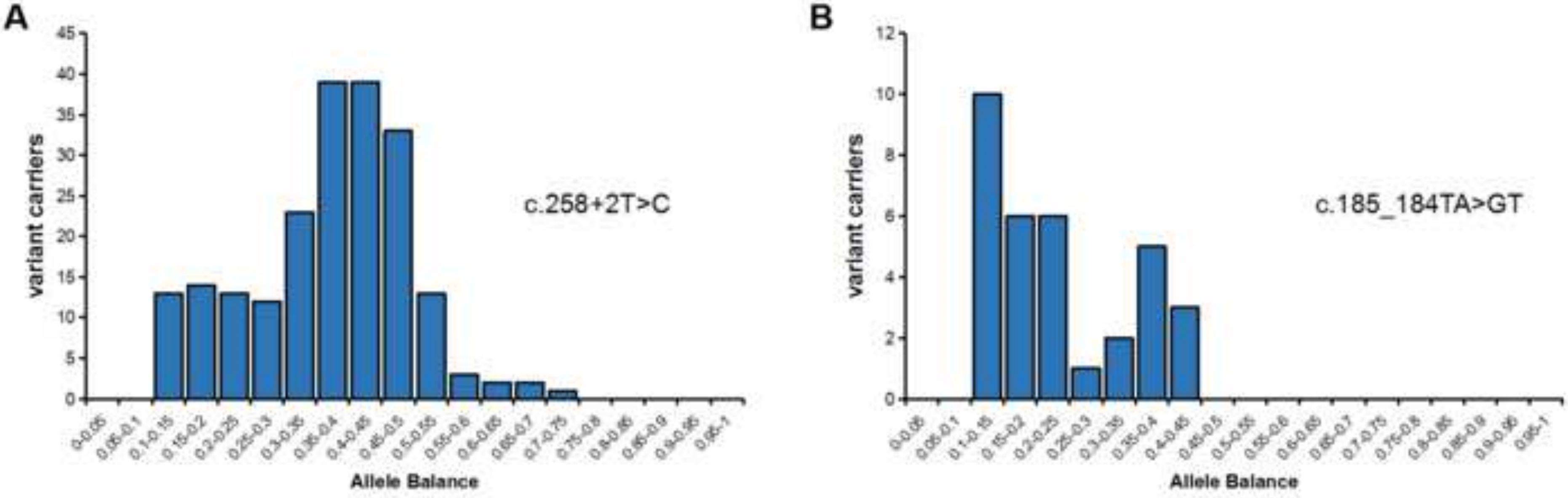

Figure 3

Click here to access/download;Figure;file-3.tif

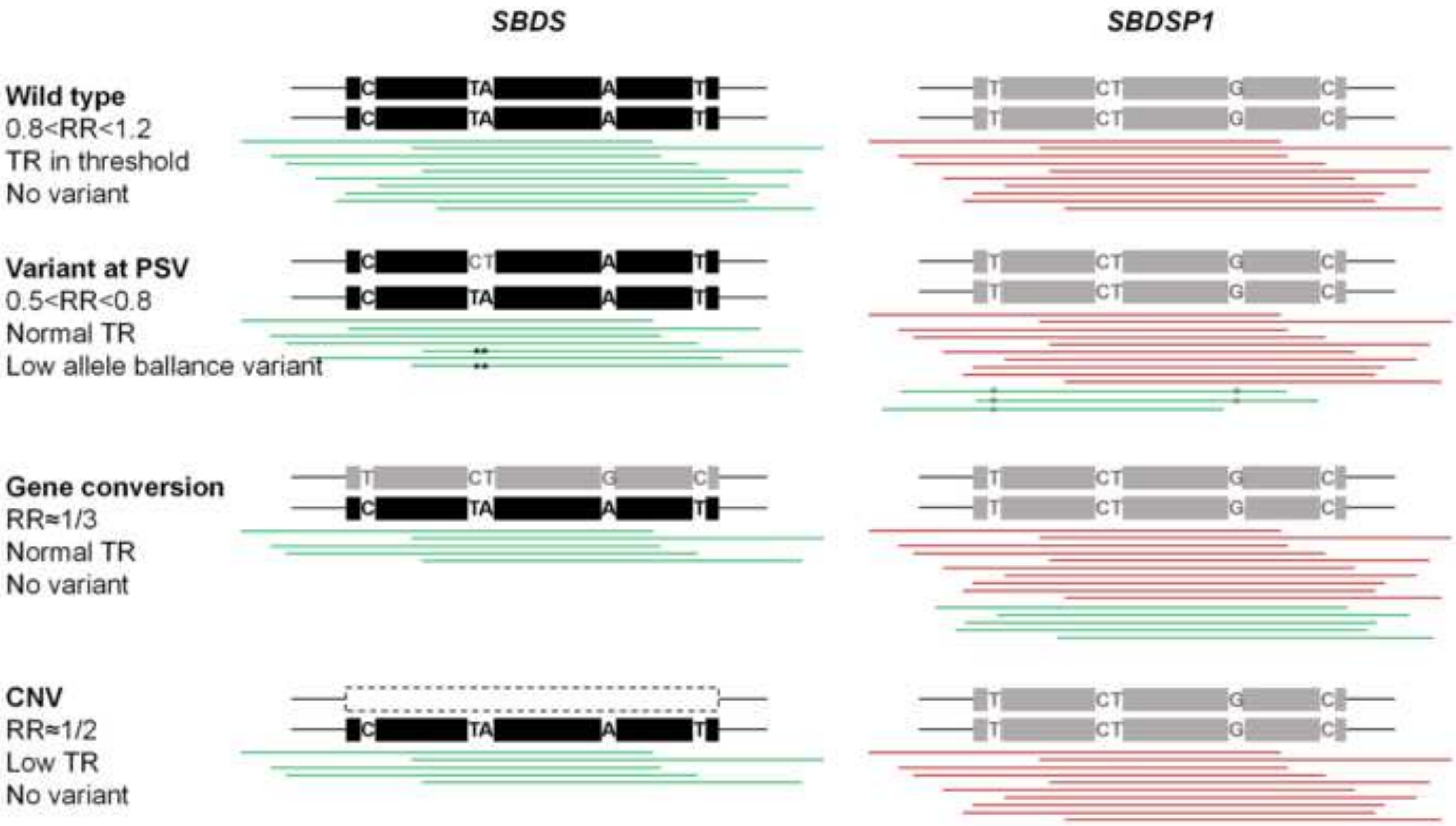

Figure 4

[Click here to access/download;Figure;file-4.tif](#)

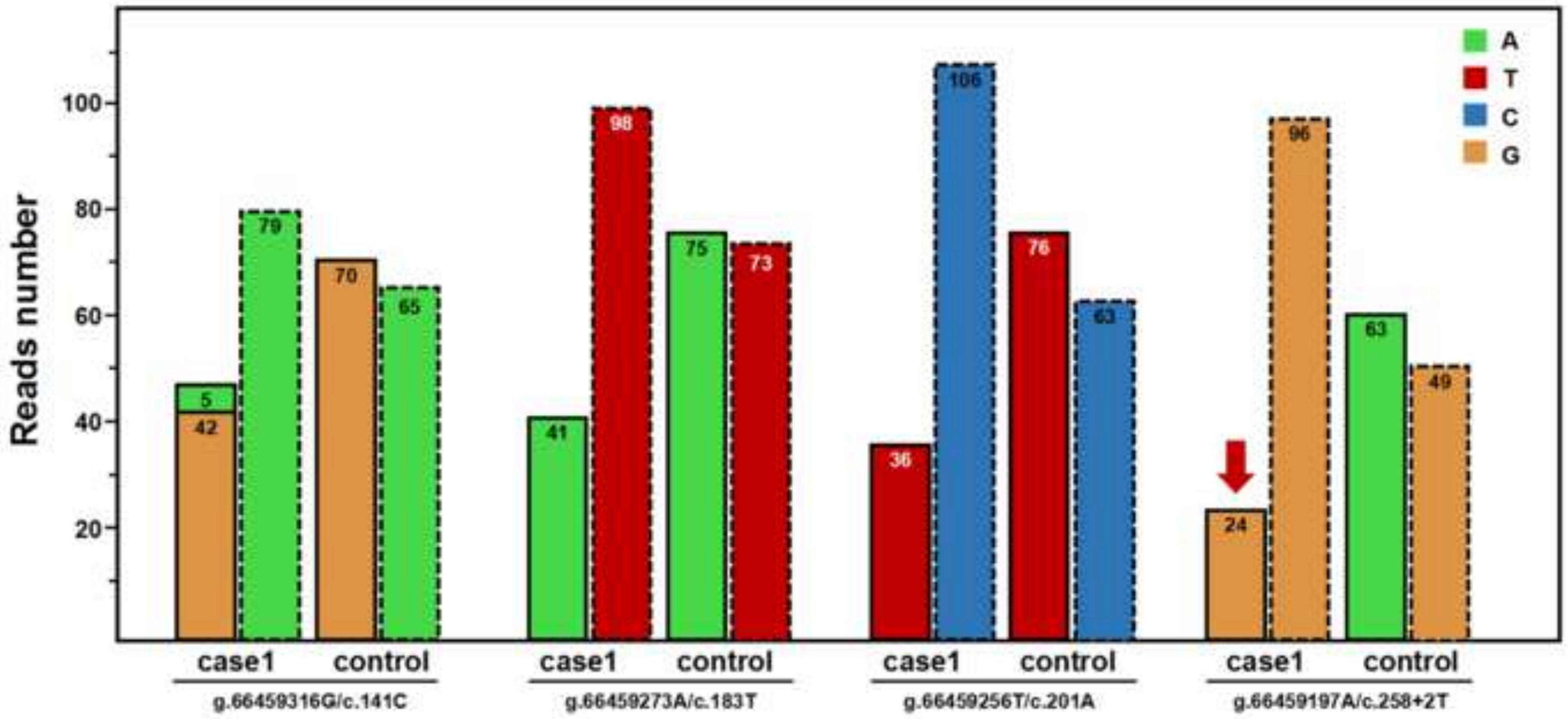

Figure 5 Click here to access/download;Figure;file-5.tif

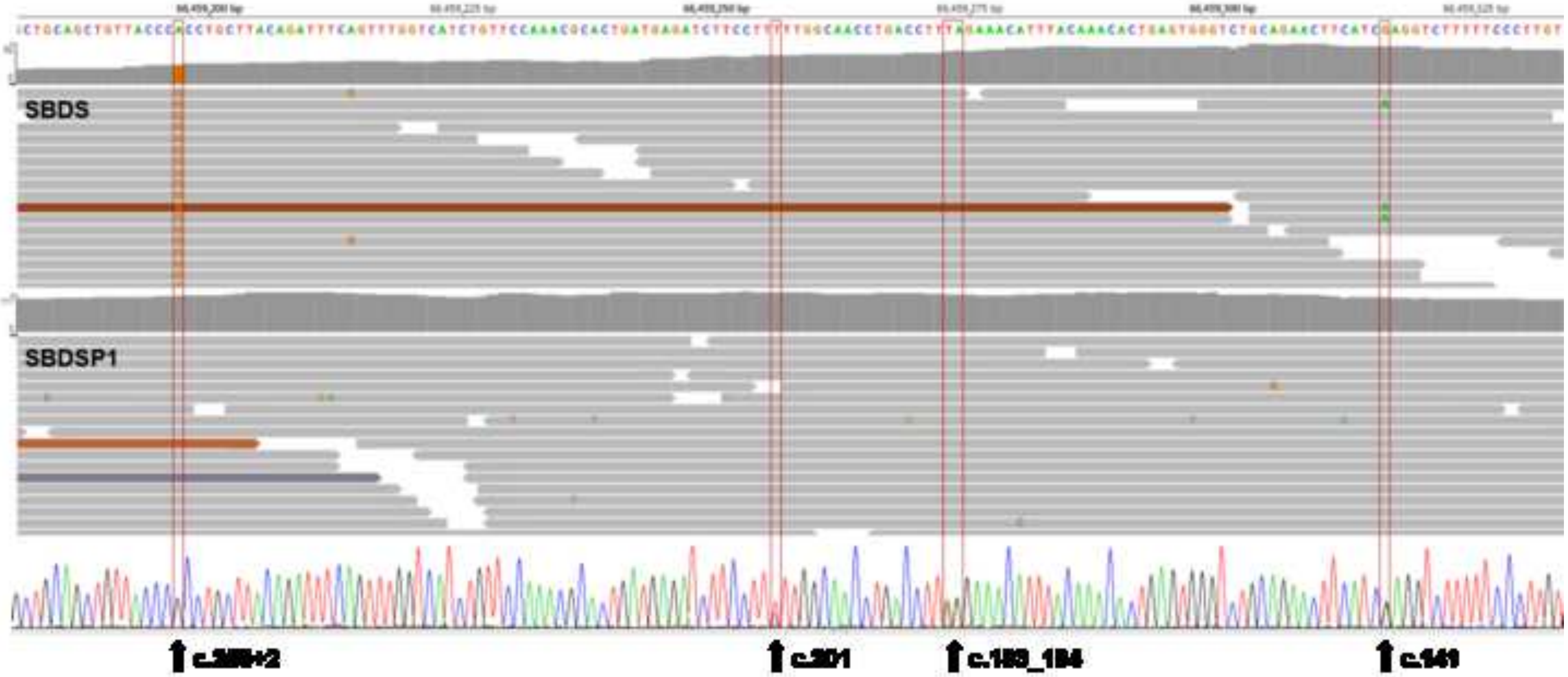

Figure 6

Click here to access/download;Figure;file-6.tif

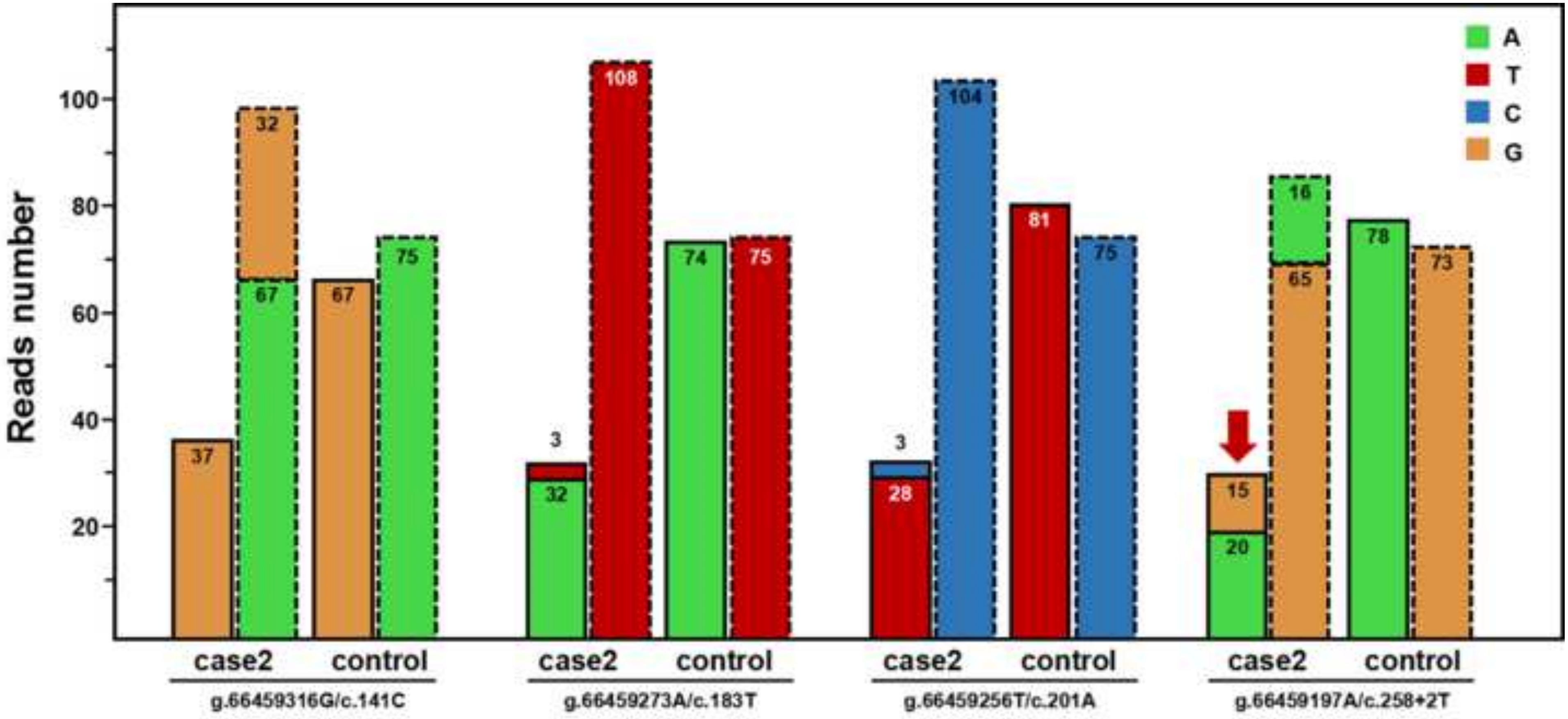

Figure 7

Click here to access/download;Figure;file-7.tif

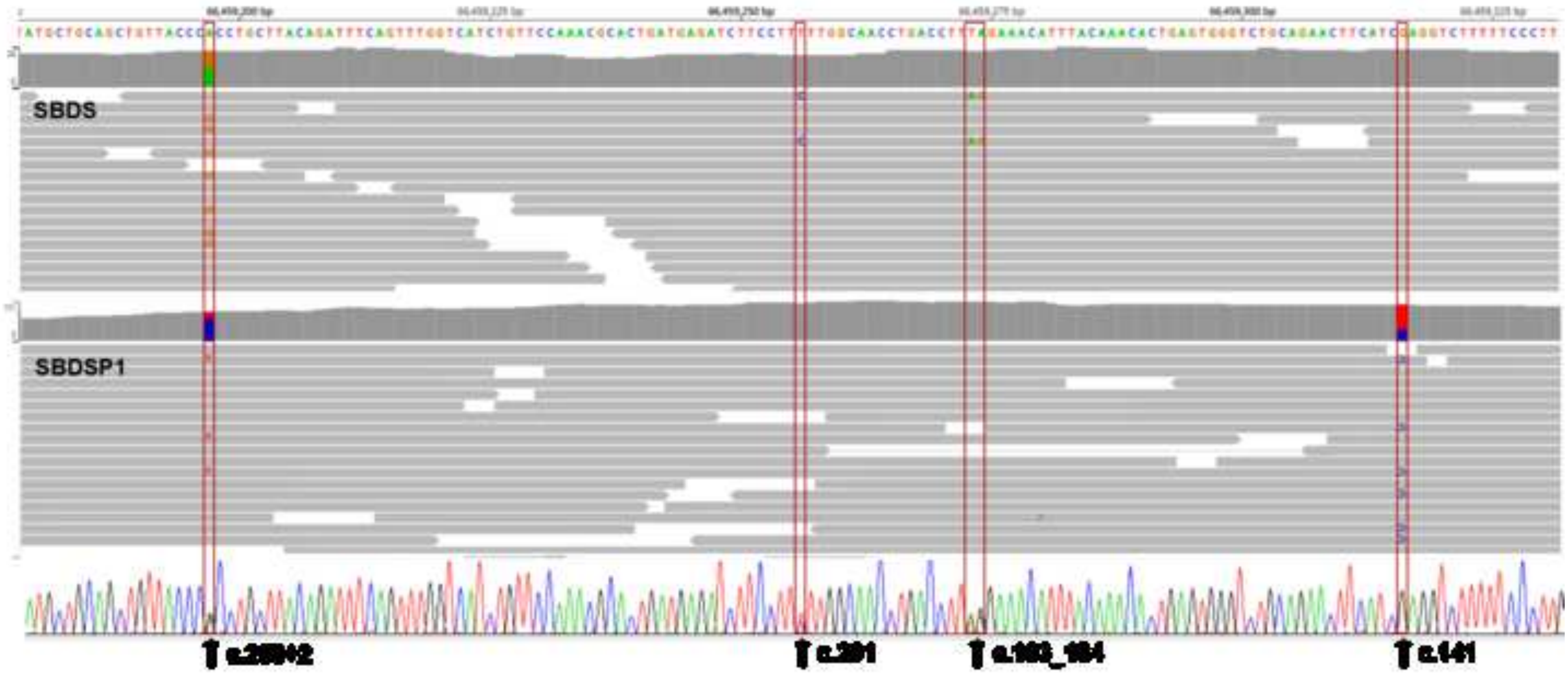

Figure 8

Click here to access/download;Figure;file-8.tif

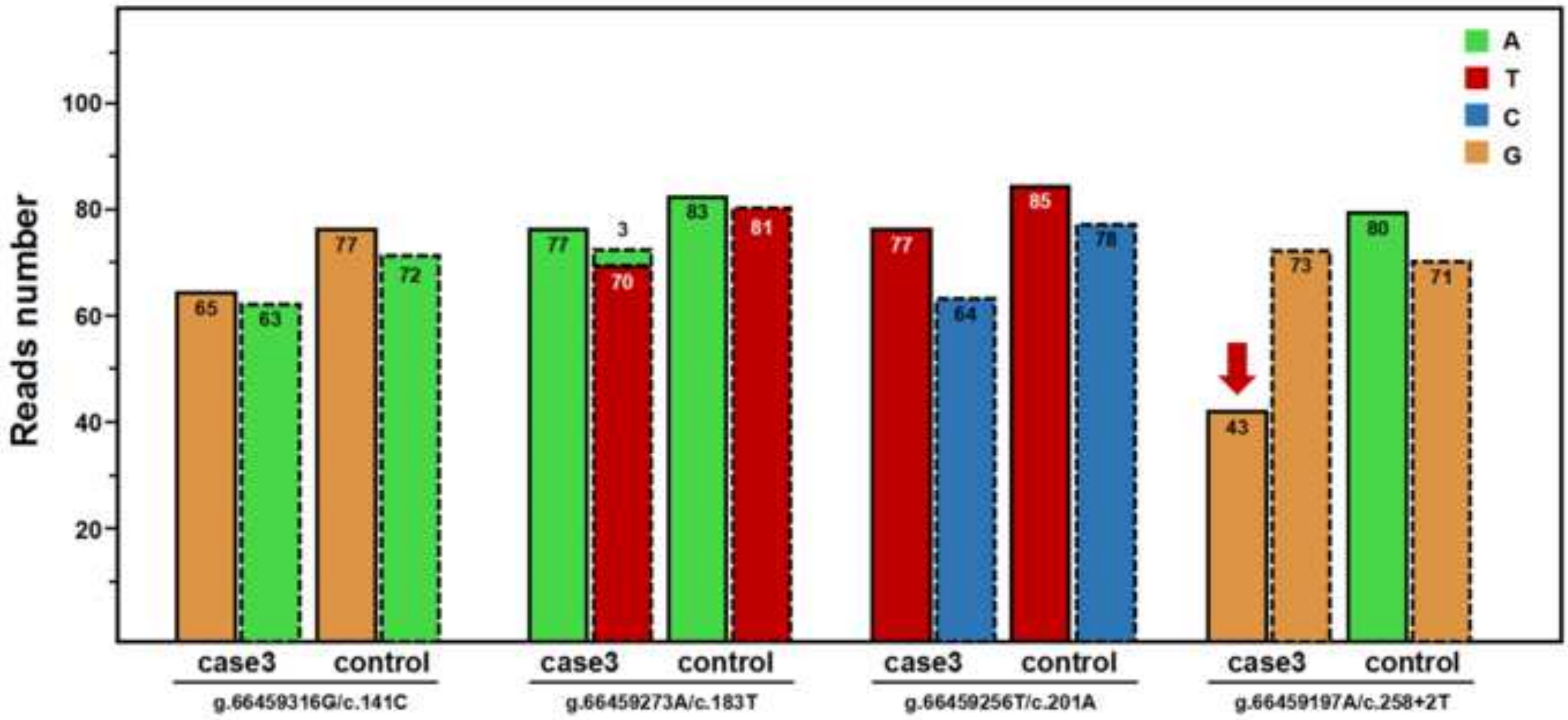

Figure 9

Click here to access/download;Figure;file-9.tif

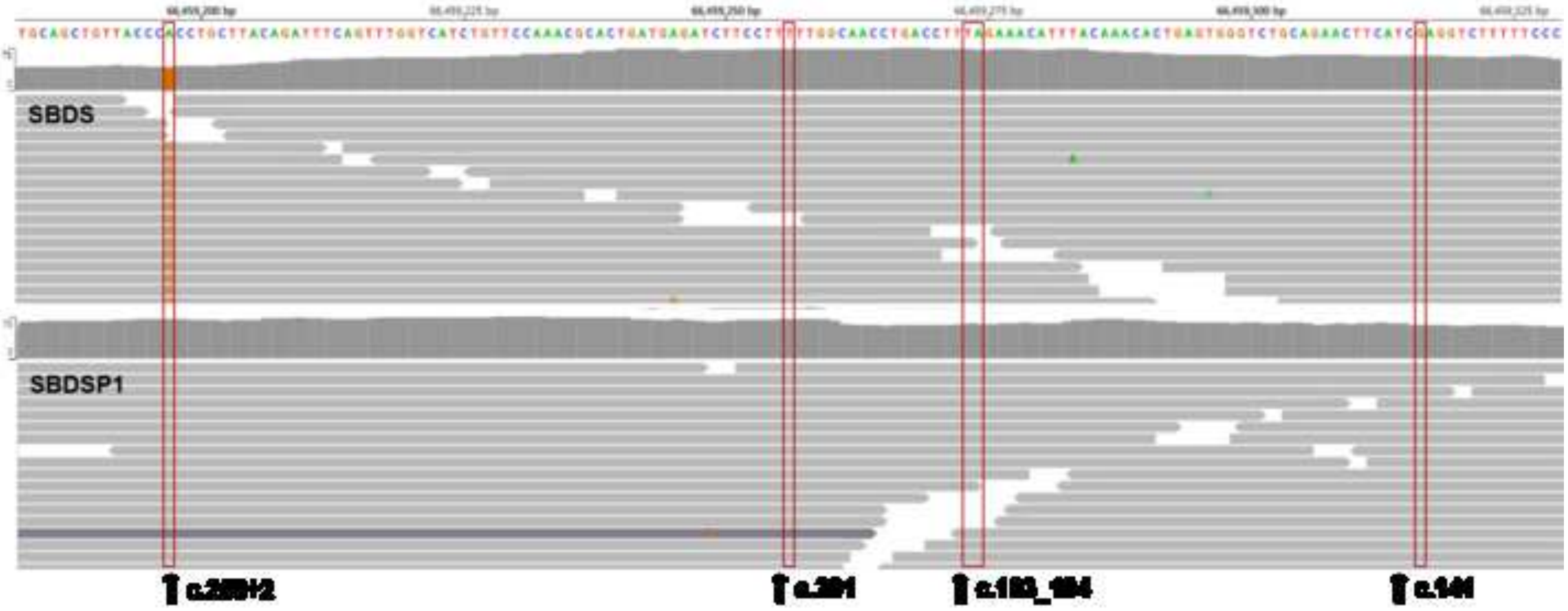

Figure 10

Click here to access/download;Figure;file-10.tif

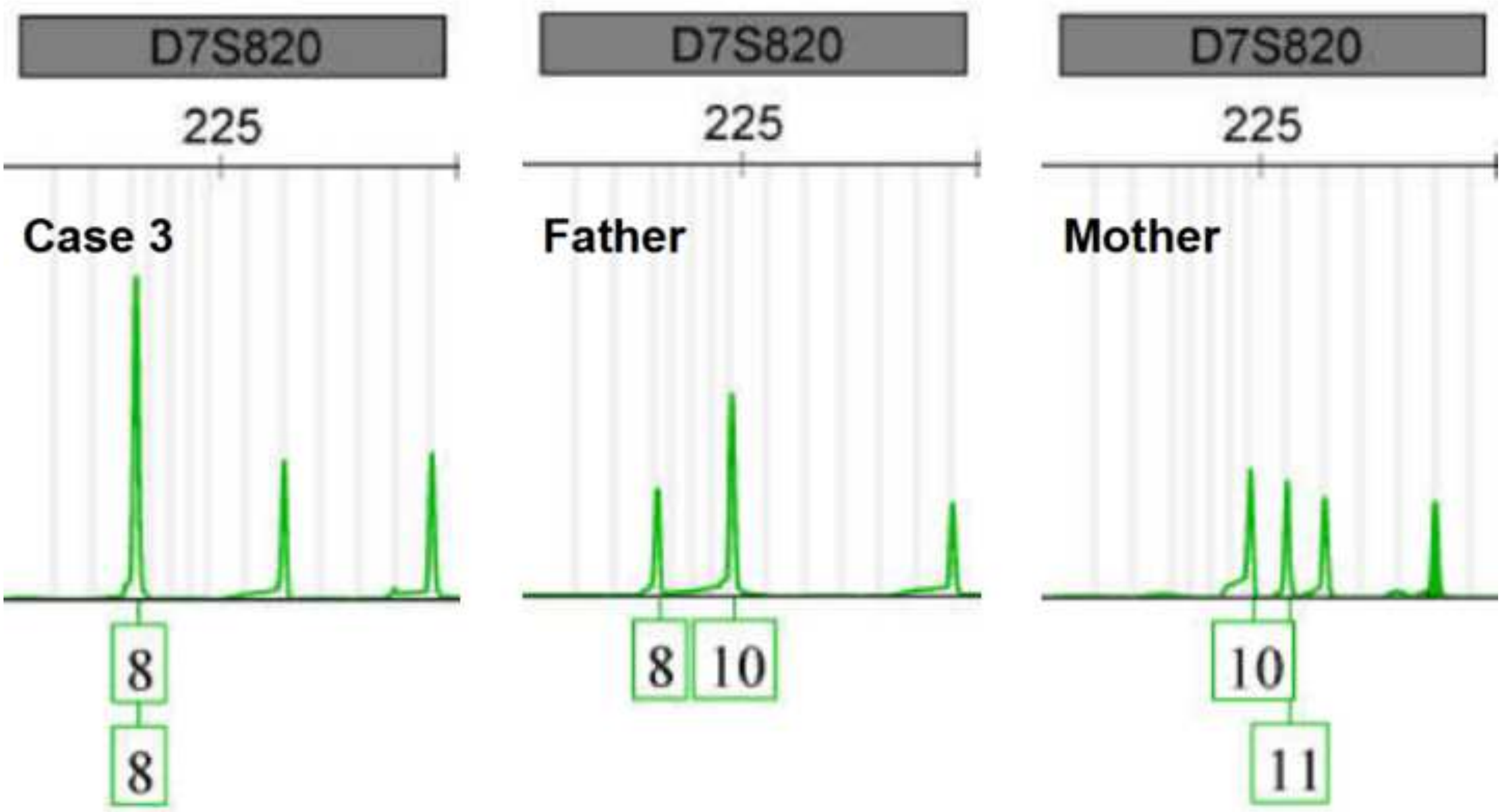

Supplemental Table 1

Click here to access/download
Supporting Information
supp-1.xlsx

Supplemental Table 2

Click here to access/download
Supporting Information
supp-2.docx
