# Supplementary material for: Improved detection of SBDS gene mutation by a new method of next-generation sequencing analysis based on the Chinese mutation spectrum
Source: PLoS One. 2022 Dec 13;17(12):e0269029. doi: 10.1371/journal.pone.0269029 (PMC9747038; doi:10.1371/journal.pone.0269029)
Supplement: Attachment — Submitted filename: Response to Reviewers.docx [file pone.0269029.s004.docx]

Dear Editors and Reviewers,

Thank you for your letter and the reviewers’ comments concerning our manuscript entitled “Improved detection of SBDS gene mutation by a new method of next-generation sequencing analysis based on the Chinese mutation spectrum” (PONE-D-21-31295). Those comments are valuable and very helpful. We have read through comments carefully and have made corrections. Based on the instructions provided in your letter, we uploaded the file of the revised manuscript. Accordingly, we have uploaded a copy of the original manuscript with all the changes highlighted by using the track changes mode in MS Word. The main corrections in the paper and the responds to the reviewer’s comments are as flowing:

**Response to reviewer #1:**

“The NGS variation data analysis method based on the reading depth analysis of SBDS and its pseudogene PSV loci proposed in this paper can effectively overcome the problem of dislocation in SBDSNGS data analysis and improve the detection rate of SBDS pathogenic variation. This method is based on the mutation spectrum of Chinese SBDS, and has certain clinical significance for more effective molecular diagnosis, mutation spectrum study and large-scale carrier screening of SDS.”

**Response:** Thank you for your summary. We really appreciate your efforts in reviewing our manuscript.

**Response to reviewer #2:**

“Overall the manuscript is interesting and valuable to assist NGS data analysis. However, my major criticize is the fact that authors claim a creation of a new method and a poor description of it is made. Thus making almost impossible to replicate it. Authors should include more details of the new method created. Further, a discussion regarding the applicability of this method to other diseases or populations is also important.”

**Response:** Thank you for your summary. We really appreciate your efforts in reviewing our manuscript. We have revised the manuscript accordingly. Our point-by-point responses are detailed below.

**Q1:** Authors should include more details of the new method created.

**Response:** We apologize for not describing the method clearer. we have added a more detailed interpretation and a flow diagram (Please see Figure3) to describe the procedure of our strategy.

**Q2:** A discussion regarding the applicability of this method to other diseases or populations is also important.

**Response:** Thank you for the suggestion. As suggested by reviewer, we have added the suggested content to the manuscript. Please see “4 Conclusion”

**Q3:** “How this is done? ”– the comment on “The average read-depths of each exon of *SBDS* and the corresponding region of *SBDSP1* were extracted to estimate the frequency of conversion between *SBDS* and *SBDSP1*.”

**Response:** Thank you for the suggestion. We have added the information required. Please see “Whole-Exome Sequencing” section in “2 Materials and Methods”.

**Q4:** “How is the processing procedure?”; “where is the mathematical model?”; “So, the mutations previously referred were from which gene?”; “so, this were not mutations? I'm confused”—the comments on “SBDS NGS analysis strategy”.

**Response:** We apologize for not describing the strategy clearer. we have added a more detailed interpretation and a flow diagram (Please see Figure3) to describe the procedure of our strategy.

Sincerely.

Kaiyu Wang
